# Carbolic Acid as an Anæsthetic

**Published:** 1873-12

**Authors:** Andrew H. Smith


### ARTICLE XI.

### *Carbolic Acid as an Anæsthetic.*

BY ANDREW H. SMITH, M. D.

Dr. Andrew H. Smith reported to the Medical Society of the County of New York, April 22d, 1872, some experiments made upon himself, which fully confirm the statement of Dr. J. H. Bill, as to the local anæsthetic power of carbolic acid.

"In my first experiment," says Dr. Smith, "I painted a spot on the forearm, about an inch in diameter, with carbolic acid of about the strength of 85 per cent. For about a minute there was a slight burning sensation after which the integument became entirely insensible, the cuticle being whitened and shriveled, and the spot slightly elevated. I then with a scalpel made an incision about half an inch in length through the whole thickness of the integument. This was done without even feeling the contact of the knife.

The capillary circulation seemed not to be materially interfered with, as the blood flowed as freely as it would from a similar wound under ordinary circumstances. The reparative process was also not impaired, cohesion taking place immediately. Three hours after the application of the acid a needle could be thrust freely into the skin without causing pain.

"In the second experiment carbolic acid was applied as before, and ten minutes after a fly blister was placed upon the spot. The blister remained eight and a half hours without causing any pain, and without producing vesication.

"In two instances I have applied the acid previous to incising a whitlow. The operation was almost painless, but, as the whitlow was in each case of the superficial variety, the test was not entirely conclusive.

"Inhaled in the form of spray, I have found the acid very useful in allaying irritation of the bronchial mucous membrane; coughs which have resisted all ordinary treatment have been immediately relieved, and in the course of two or three days entirely removed.

"I would suggest the use of carbolic acid as a revulsive, in cases in which a continuous impression is desired. While causing but little suffering, it produces an intense hyperæmia of the skin, which persists for eight or ten days, and is followed by the desquamation of the cuticle."—*New York Medical Journal.*

